# *ALKBH5* promotes hepatocellular carcinoma cell proliferation, migration, and invasion by regulating *TTI1* expression

**DOI:** 10.17305/bb.2024.10247

**Published:** 2024-10-01

**Authors:** Qimeng Chang, Xiang Zhou, Huarong Mao, Jinfeng Feng, Xubo Wu, Ziping Zhang, Zhiqiu Hu

**Affiliations:** 1 Department of Hepatobiliary-Pancreatic Surgery, Minhang Hospital, Fudan University, Shanghai, China; 2 Institute of Fudan-Minhang Academic Health System, Minhang Hospital, Fudan University, Shanghai, China

**Keywords:** Hepatocellular carcinoma (HCC), ALKBH5, TTI1, proliferation, migration, invasion

## Abstract

Understanding the function of AlkB homolog 5, RNA demethylase (*ALKBH5*) in hepatocellular carcinoma (HCC) holds promise for unraveling new therapeutic strategies for combating this malignancy. The objective of this research was to investigate the potential mechanisms of *ALKBH5* in HCC. We used The Cancer Genome Atlas (TCGA), Kruskal–Wallis method, and Kaplan–Meier (KM) survival analysis to study the expression of *ALKBH5* and its correlation with clinical factors in HCC. In vitro experiments verified the expression of *ALKBH5* and its effect on HCC cell phenotype. We screened differentially expressed genes (DEGs) from HCC patients associated with *ALKBH5*. Through this screening, we identified the downstream gene *TTI1* which is associated with *ALKBH5,* and investigated its function using gene expression profiling interaction analysis (GEPIA) along with univariate Cox proportional hazards regression analysis. Finally, we analyzed the functions of *ALKBH5* and *TTI1* in HCC cells. Across numerous pan-cancer types, we observed significant overexpression of *ALKBH5*. In vitro experiments confirmed *ALKBH5* as an oncogene in HCC, with its knockdown leading to suppressed cell proliferation, migration, and invasion. Bioinformatics analyses also demonstrated a significant positive correlation between *ALKBH5* and *TTI1*. *TTI1*, highly expressed in cells, showed promising prognostic ability for patients. Further experiments confirmed that suppressing *TTI1* impeded cell growth and movement, with this effect partially offset by increased *ALKBH5* expression. Conversely, promoting these cellular processes was observed with *TTI1* overexpression, but was dampened by decreased *ALKBH5* expression. In conclusion, our findings suggest that *ALKBH5* may influence the proliferation, migration, and invasion of HCC by modulating *TTI1* expression, providing a new direction for treating HCC.

## Introduction

Liver carcinoma, characterized as a malignant neoplasm, has been extensively linked with risk factors, such as excessive alcohol consumption, viral hepatitis, consumption of mold-contaminated food, and genetic predispositions [1]. This disease can be histopathologically subdivided into two principal categories: hepatocellular carcinoma (HCC) and intrahepatic cholangiocarcinoma (iCCA) [2]. HCC, accounting for 90% of primary liver cancer cases, represents the predominant histological subtype of this malignancy. Key contributory factors implicated in HCC include aflatoxin exposure, obesity, epigenetic alterations, heredity, infection with the hepatitis C virus, tobacco smoking, chronic hepatitis B, and diabetes [3]. Currently, among methods for HCC treatment, transplantation is the most effective [4]. Interventional therapy, ablation therapy, chemoradiotherapy, and targeted therapy can be applied to patients with unresectable liver carcinoma [5, 6]. Regrettably, despite these interventions, the prognosis for HCC remains dismal due to high rates of metastasis and recurrence [7]. This highlights the necessity of uncovering the molecular processes driving HCC progression and pinpointing new therapeutic targets.

The AlkB homolog 5, RNA demethylase (*ALKBH5*), also known as ABH5, OFOXD, or OFOXD1, is implicated in the biological cascades of various neoplasms [8]. Notably, Guo et al. showed that *ALKBH5* could curb pancreatic carcinoma progression through PER1 activation in an m6A-YTHDF2-mediated pathway. This finding reveals that *ALKBH5* inhibits pancreatic cancer by regulating the post-transcriptional activation of PER1 through modulation of m6A modifications [9]. And there have been previous studies describing the role of *ALKBH5* in liver cancer. For example, *ALKBH5* may be a key effector associated with macrophage M2 polarization. The *ALKBH5*/SOX4 axis promotes HCC stem cell properties through activation of the Sonic Hedgehog (SHH) signaling pathway [10]. Circ-CCT3 is subjected to *ALKBH5*- and METTL3-mediated m6A modification and promotes HCC development through the miR-378a-3p-FLT1 axis [11]. *ALKBH5* acts as a dual role of a microenvironmental regulator and a radiosensitization target, mediating monocyte recruitment and M2 polarization and creating positive feedback to reduce HCC radiosensitivity [12]. *ALKBH5*-mediated lincRNA affects HCC growth and metastasis requiring methylation [13]. *ALKBH5* promotes HCC growth, metastasis, and macrophage recruitment through the *ALKBH5*/MAP3K8 axis [14]. It has been reported not only in cancer but also in diseases such as another literature by Shen et al. [15] established that *ALKBH5* selectively augments the incidence of acute myeloid leukemia (AML) and the self-renewal of carcinoma stem cells. Moreover, *ALKBH5* has been reported to function as a tumor promoter in AML by post-transcriptionally modulating pivotal targets, such as TACC3, an oncogene related to prognosis in a broad spectrum of carcinomas [16–19]. Together, these observations underscore the central function of *ALKBH5* in the pathogenesis of leukemia and the self-renewal of leukemia stem cells/leukemia-initiating cells (LSC/LIC), thereby highlighting the role of *ALKBH5*/N6-methyladenine (m6A) axis for therapeutic potential. These revelations pave the way for further exploration of the roles played by *ALKBH5* in the pathogenesis of diverse human carcinomas.

Our research attempted to illuminate the role of *ALKBH5* in HCC, utilizing an integrative approach of bioinformatics analysis and cellular experiments. In parallel, we worked to uncover the precise mechanism by which *ALKBH5* and HCC are interconnected. It is anticipated that our findings may provide new insights and pave the way for advances in therapeutic intervention, diagnosis, and prognostic assessment of HCC patients.

## Materials and methods

### Evaluation of *ALKBH5* expression levels across pan-cancers

To assess the expression levels of *ALKBH5* across a range of cancer types, we utilized data procured from The Cancer Genome Atlas (TCGA; https://tcga-data.nci.nih.gov/tcga) and the Genotype-Tissue Expression (GTEx; https://www.gtexportal.org/home/) database. The TCGA includes a large multidimensional map of key genomic changes in various cancers. On the other hand, the GTEx project provides a valuable resource that facilitates the study of human gene expression and regulation in different tissue types. By integrating these resources, we conducted a pan-cancer study exploring *ALKBH5* expression across 33 cancer types, in comparison to normal tissues. The analysis was executed using R software, offering a comprehensive visual depiction of *ALKBH5* expression distribution.

### Correlation analysis between *ALKBH5* expression and HCC clinical parameters

HCC samples were obtained from the TCGA for comprehensive surveys. We used the Kruskal–Wallis method to analyze the differential expression of *ALKBH5* in the context of multiple clinical parameters of HCC, including nodal status (node), metastatic status (metastasis), pT stage, pTNM stage, grade, hepatitis B virus (HBV), and hepatitis C virus (HCV). This nonparametric statistical test allowed us to compare expression levels across numerous independent groups. Subsequently, to graphically illustrate the mutual relationships and transitions among these clinical parameters, *ALKBH5* expression, and patient status, we employed the “ggplot2” package in R to generate an informative and visual Sankey diagram. This diagram serves to provide an intuitive understanding of the interplay among these key aspects.

### Analysis of functional pathways in differentially expressed genes (DEGs)

According to the expression of *ALKBH5* in HCC, we divided the TCGA-HCC patient data into *ALKBH5*-high and *ALKBH5*-low cohorts for screening DEGs. Subsequently, we screened for upregulated DEGs (*P* < 0.05 and fold change (FC) > 1.5) and downregulated DEGs (*P* < 0.05 and FC < 0.67) using the Limma package in R software. Next, to further elucidate the biological roles of the DEGs, we performed the Kyoto Encyclopedia of Genes and Genomes (KEGG) pathway enrichment analysis using the Enrichr tool (https://maayanlab.cloud/Enrichr/). Findings with a *P* value below 0.05 were deemed to be of statistical relevance.

### Analysis of genes associated with prognosis of HCC

We performed a progression-free survival (PFS) analysis of 100 upregulated and 100 downregulated DEGs. Statistical tests for differences in PFS between low and high expression groups were performed using the log-rank test in Kaplan–Meier (KM) survival analysis, and hazard ratios (HRs), 95% confidence intervals (CIs), and *P* values were subsequently generated. From this survival analysis, we focused on genes with *P* values less than 0.05, indicating statistical significance. To gain a comprehensive understanding of the protein–protein interaction (PPI) associated with these genes, we exploited the potential of the proteins encoded by statistically significant genes. We used the Search Tool for the Retrieval of Interacting Genes (STRING) database (https://string-db.org/) as the primary platform for interactive data. Additionally, network visualization and analysis were performed using Cytoscape software. Subsequently, for the genes that showed statistical significance in the survival analysis, we performed a gene correlation analysis, the results of which were visualized by the R package “heatmap.”

### Screening of candidate genes in the prognostic model

We utilized Least Absolute Shrinkage and Selection Operator (LASSO) regression, implemented through the “glmnet” package in R, to construct a polygenic signature for the prognostic prediction of HCC using genes that displayed significant *P* values. To ensure the reliability and objectivity of the analysis, ten-fold cross-validation was conducted to select the optimal lambda (λ) value that corresponds to the smallest error fraction. Subsequently, HCC patients were divided into high-risk and low-risk groups, and their survival time, risk score, as well as survival status were obtained from the selected dataset. The z-scores of six gene expressions (*TTI1*, *ACIN1*, *ADNP*, *CFHR3*, *SPP2*, and *HGFAC*) in these patients were displayed by heat map. Ultimately, the prognostic implications of the high-risk and low-risk groups were substantiated through KM survival analysis. To identify significant variations in PFS probability between the two groups, a log-rank test was carried out. Additionally, time-dependent receiver operating characteristic (ROC) analysis for 1-year, 3-year, and 5-year survival forecasts was used to assess the prognostic performance of the risk model. The area under the curve (AUC) method was used to determine the prediction accuracy of the model.

### Correlation analysis between *ALKBH5* and candidate target genes

As a newly developed interactive web server, Gene Expression Profiling Interactive Analysis (GEPIA) offers features that are customized, which are inclusive of patient survival analysis and gene detection. In this study, we employed GEPIA to investigate the relationship between *ALKBH5* and potential target genes. Through the computation of correlation coefficients, we identified a key gene displaying the strongest correlation with *ALKBH5* and HCC. When *P* < 0.05, the results were deemed statistically significant.

### Construction and verification of the predictive nomogram with *TTI1*

Initially, we evaluated the expression level of *TTI1* in HCC samples utilizing the Wilcoxon test. Subsequently, the prognostic significance of *TTI1* was compared with clinical parameters, such as pM stage, pT stage, pTNM stage, age, and grade, through a univariate Cox proportional hazards regression analysis. To ascertain if *TTI1* could function as an independent prognostic factor for risk stratification in HCC patients, a multivariate Cox proportional hazards regression analysis was executed. This analysis incorporated additional clinical parameters that exhibited statistical significance (*P* < 0.05) in the univariate Cox regression model. Based on the independent prognostic factors identified from the preceding analyses, a composite nomogram was constructed via the “rms” package in R. This predictive tool was designed to forecast 1-, 3-, and 5-year survival probabilities, and its performance was subsequently assessed through a calibration curve.

### Cell culture and transfection

We procured LO2 (normal liver cell), MHCC-97L, MHCC-97H, and SNU387 (HCC cell lines) from the American Type Culture Collection (ATCC, Manassas, VA, USA). LO2 cells were maintained in IMDM-RMPI supplemented with 1% penicillin–streptomycin and 10% fetal bovine serum (FBS), while MHCC-97L, MHCC-97H, and SNU387 cells were cultured in DMEM, fortified with the same supplements. All cell lines were incubated at 37 ^∘^C in an environment containing 5% CO_2_. For cell transfection, we acquired si-*ALKBH5*#1, si-*ALKBH5*#2, si-*ALKBH5*#3, over-*ALKBH5*, si-*TTI1*#1, si-*TTI1*#2, si-*TTI1*#3, over-*TTI1*, along with their negative controls (over-NC and si-NC) from the Shanghai Biotech Company, Gemma Gene. Transfection was carried out using Lipofectamine 3000 (Invitrogen), and the efficiency of this process was evaluated under a fluorescence microscope.

### RNA extraction and quantitative real-time polymerase chain reaction (qRT-PCR) analysis

TRIzol (Invitrogen) was used to extract the total RNA from the cells. The RevertAid First Strand cDNA Synthesis Kit from Invitrogen was used to create complementary DNA (cDNA). qRT-PCR was carried out using an Applied Biosystems 7900 real-time PCR System and the SYBR Green PCR Master Mix. The 2^−ΔΔCt^ technique was used to compare the relative expression levels of *ALKBH5* and *TTI1*, with *GAPDH* acting as an internal control. The following primers were used in this experiment: *ALKBH5* (forward: 5′-CGGCGAAGGCTACACTTACG-3′; reverse: 5′-CCACCAGCTTTTGGATCACCA-3′), *TTI1* (forward: 5′-CCACAGCTGAAGACATCGAA-3′; reverse: 5′-ACATCTGGACGGGTGTCATT-3′) and *GAPDH* (forward: 5′-CAAGGTCATCCATGACAACTTTG-3′; reverse: 5′- GGGCCATCCACAGTCTTCT-3′).

### Western Blotting (WB) assay

ALKBH5 and TTI1 protein expression in HCC cell lines were evaluated using a WB assay. Cells underwent lysis and protein extraction with the RIPA buffer containing a protease inhibitor cocktail. Protein concentrations were determined via a bicinchoninic acid (BCA) assay kit. Proteins were then subjected to sodium dodecyl sulfate polyacrylamide gel electrophoresis (SDS-PAGE) and transferred to polyvinylidene fluoride (PVDF) membranes. Post-transfer, membranes were blocked using 5% nonfat milk for non-specific binding prevention. They were then probed overnight at 4 ^∘^C with primary antibodies for ALKBH5 (1:1000) and TTI1 (1:500). GAPDH (1:1000) served as the control. After washing, membranes were exposed to HRP-linked secondary antibodies (1:5000) for an hour. Protein bands were detected with an enhanced chemiluminescence (ECL) system and quantified by densitometry.

### Assay for cell proliferation

Cell Counting Kit-8 (CCK-8) (Shiga Doto Molecular Technology, Japan) was applied to measure cell proliferation. First, we plated 2×10^4^ of the transfected SNU387 cells in a 96-well plate in triplicate. Then, 10 µL of CCK-8 solution per well was added into cells for the indicated time. After 24, 48, 72, 96, and 120 h, we finished measuring the optical density (OD) value of the cells at 450 nm via iMark Microplate Reader (Bio-Rad) for plotting the cell proliferation curve.

**Figure 1.**
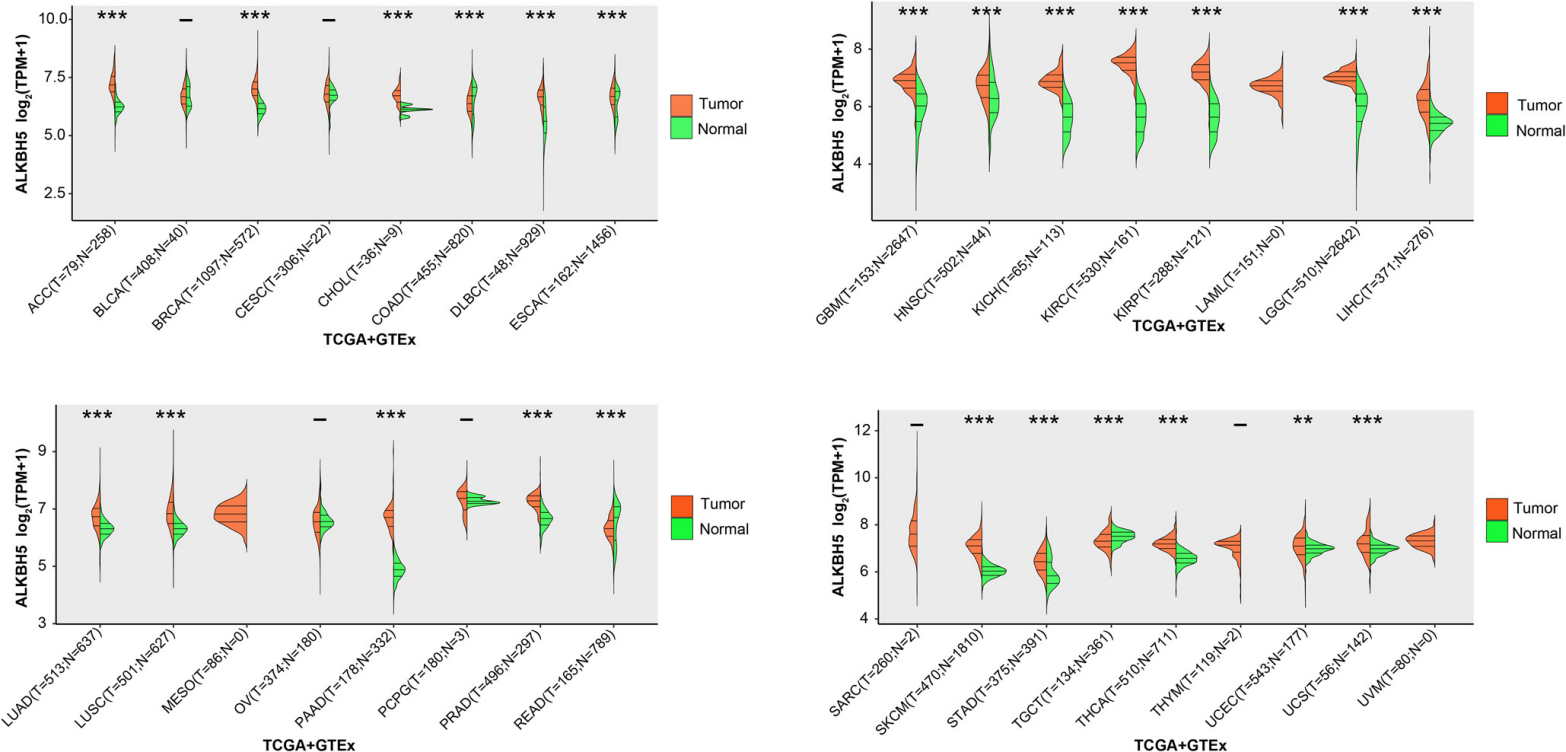
**Expression verification of *ALKBH5* in 33 tumors.** The horizontal axis represents the tumor and normal samples in the TCGA and GTEx databases, the vertical axis represents the expression distribution of *ALKBH5*, and different colors represent different groups. **P < 0.01, ***P < 0.001. TCGA: The Cancer Genome Atlas; GTEx: Genotype-Tissue Expression.

### Transwell assay

The invasive and migratory abilities of cells were determined utilizing a Transwell chamber assay (Corning). The upper chamber was covered with 100 L of Matrigel (Corning) and allowed to set up for an hour at 37 ^∘^C in preparation for the invasion assay. Subsequently, a 0.5 µL cell suspension was added to the Matrigel-coated chamber, which was filled with DMEM medium devoid of serum. After the invasion and migration phases are complete, cells are stained with DAPI to reveal nuclei. High-resolution images were captured after a 20 min staining period, utilizing an Olympus BX53 upright microscope equipped with a digital camera. The migration assay was performed in a similar manner, except the Matrigel coating step was omitted. Each of these experimental steps was independently replicated three times to ensure the accuracy and reproducibility of the findings.

### Ethical statement

This study used publicly available datasets and therefore was exempt from the traditional ethics approval process, making a formal ethics statement not applicable to this study.

### Statistical analysis

R software was used for all statistical analyses. The Kruskal–Wallis or Wilcoxon test was used to analyze differences in expression among groups. The log-rank test was used to examine KM survival curves. Correlation coefficients were used to examine the relationship between *ALKBH5* and clinical factors or prospective target genes. The prognostic model was built using LASSO regression with ten-fold cross-validation. To investigate the prognostic value, univariate and multivariate Cox proportional hazard regression models were used. The in vitro experiments were conducted three times and the results are reported as mean ± standard deviation. Statistical significance was defined as *P* < 0.05.

## Results

### Significantly high expression of *ALKBH5* in the majority of pan-cancers

We have analyzed the expression levels of *ALKBH5* across 33 different human malignancies, utilizing data from both the TCGA and GTEx databases. As illustrated in Figure 1, *ALKBH5* exhibits significant overexpression in a majority of tumor tissues when compared to their neighboring normal tissues, including breast invasive carcinoma (BRCA), adrenocortical carcinoma (ACC), cholangiocarcinoma (CHOL), HCC, etc. This pattern of differential expression implies a potential role of *ALKBH5* in the oncogenesis and progression of these tumor types, warranting further investigation into its mechanistic contributions.

### Effect of differential expression of *ALKBH5* in HCC on staging, HBV infection, and patient survival

**Figure 2.**
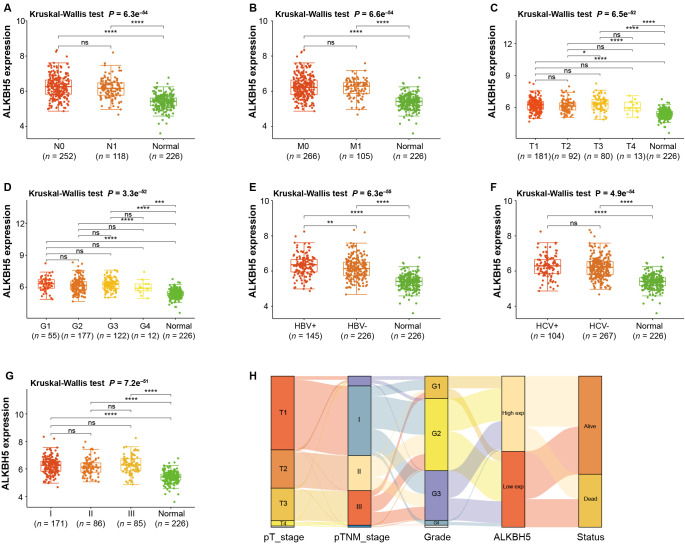
***ALKBH5* expressions in HCC patients with different clinical factors.** (A–G) Kruskal–Wallis test *ALKBH5* expression in lymph node metastasis status, distant metastasis, pT stage, grade, hepatitis B virus infection, hepatitis C virus infection, and pTNM stage. *P < 0.05, **P < 0.01, ***P < 0.001, ****P < 0.0001, ns means not statistically significant. (H) Sankey diagram. Every column stands for a characteristic variable, different colors stand for different types or stages, and the lines stand for the distribution of the same sample in different characteristic variables. HCC: Hepatocellular carcinoma; pTNM: Pathological tumor, node, metastasis.

To elucidate the role of *ALKBH5* in HCC, we scrutinized its expression profile in relation to various clinicopathological parameters (Figure 2A–2G). Notably, *ALKBH5* expression showed significant variations between T2 and T3 stages, as well as between patients with HBV+ and HBV− infection. In contrast, no substantial differences in *ALKBH5* expression were found when examined across other clinicopathological parameters. Furthermore, we observed a noteworthy interconnection between *ALKBH5* expression, clinical characteristics, and patient survival across different stages of HCC. A visual representation of these associations was provided in the form of a Sankey diagram (Figure 2H). The observed patterns suggest a potential differential role of *ALKBH5* in specific stages of tumor progression and the context of HBV infection. The association of *ALKBH5* expression with patient survival further implies its potential as a prognostic marker in HCC.

### Knockdown of *ALKBH5* inhibits growth of HCC cells in vitro

**Figure 3.**
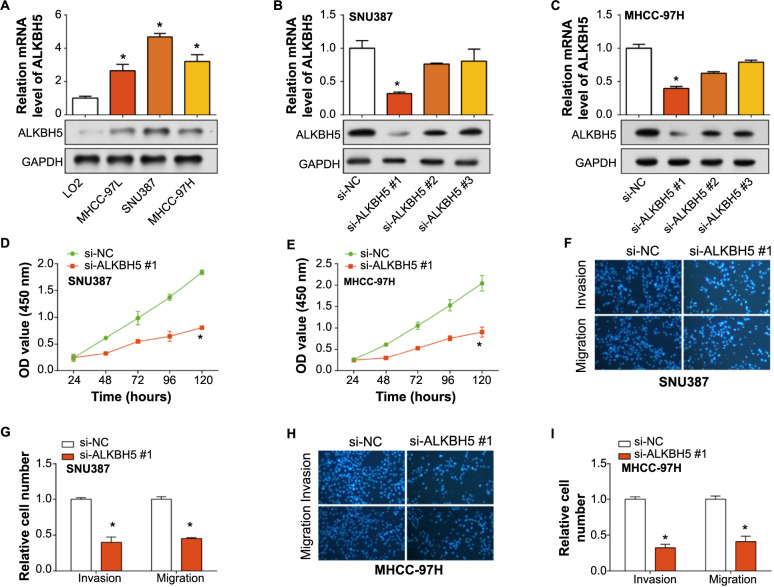
**Expression and functional analysis of ALKBH5 in normal and HCC cell lines.** (A) qRT-PCR and WB detection of ALKBH5 expression levels in normal cells and HCC cell lines; (B) qRT-PCR and WB detection of knockdown efficiency of ALKBH5 in SNU387 cells; (C) qRT-PCR and WB detection of knockdown efficiency of ALKBH5 in MHCC-97H cells; (D and E) CCK-8 detects the regulation of si-*ALKBH5*#1 on the proliferation of SNU387 and MHCC-97H cells; (F–I) Transwell detection of the regulation of si-*ALKBH5*#1 on the invasion and migration of SNU387 and MHCC-97H cells. The left panel shows magnified field views, while the right panel represents the quantified bar graphs. *P < 0.05. HCC: Hepatocellular carcinoma; WB: Western blot; CCK-8: Cell Counting Kit-8; qRT-PCR: Quantitative real-time polymerase chain reaction; OD: Optical density; si-NC: siRNA negative control.

We initially examined the expression of *ALKBH5* in HCC cell lines and normal cells. The results demonstrated an upregulation of *ALKBH5* in HCC cell lines, particularly in the SNU387 and MHCC-97H cells, prompting us to select these cell lines for subsequent investigations (Figure 3A). Next, we subjected the SNU387 and MHCC-97H cells to knockdown procedures, finding that si-*ALKBH5*#1 demonstrated the highest efficiency and was consequently selected for further experimentation (Figure 3B and 3C). Data from the CCK-8 assay revealed that the knockdown of *ALKBH5* resulted in a suppressed proliferation of SNU387 and MHCC-97H cells (Figure 3D and 3E). Furthermore, results from the Transwell assay indicated that compared with the si-NC transfected cells, SNU387 and MHCC-97H cells transfected with si-*ALKBH5*#1 exhibited significantly reduced invasion and migration capabilities (Figure 3F–3I). These results suggest a critical function of *ALKBH5* in regulating the proliferative and metastatic potential of HCC cells.

### KEGG pathway enrichment analysis on DEGs

**Figure 4.**
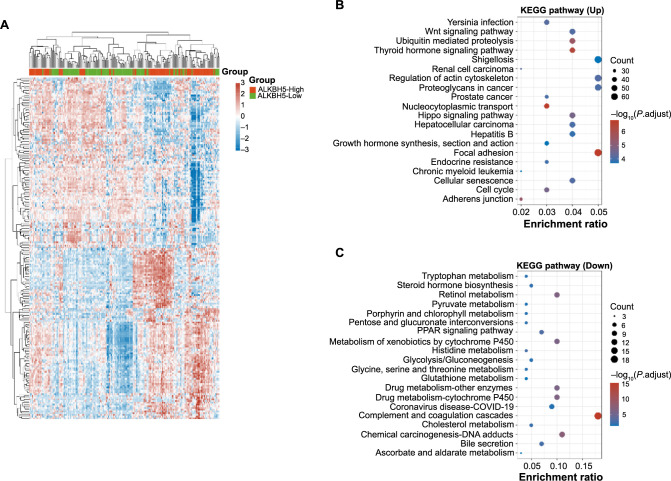
**Distribution and pathway enrichment analysis of DEGs in HCC samples based on ALKBH5 expression levels.** (A) Heat map of cluster distribution of upregulated DEGs and downregulated DEGs in HCC samples with high and low expression of ALKBH5; (B and C) Bubble plots of KEGG pathway enrichment analysis for upregulated and downregulated DEGs. Each bubble in the plot represents a different KEGG pathway, the size of the bubble corresponds to the number of DEGs associated with a particular pathway, and the color of the bubble represents the significance of the enrichment. DEG: Differentially expressed genes; HCC: Hepatocellular carcinoma; KEGG: Kyoto Encyclopedia of Genes and Genomes.

From the two groups of samples with differential expression of *ALKBH5*, we screened 3105 upregulated DEGs and 156 downregulated DEGs (Figure 4A). DEGs that were upregulated in the KEGG pathway were primarily enriched in Shigellosis, focal adhesion, regulation of actin cytoskeleton, proteoglycans in cancer, etc. (Figure 4B). DEGs that were downregulated in the KEGG pathway were primarily enriched in retinol metabolism, complement and coagulation cascades, chemical carcinogenesis-DNA adducts, etc. (Figure 4C).

### 36 key genes associated with HCC prognosis

**Figure 5.**
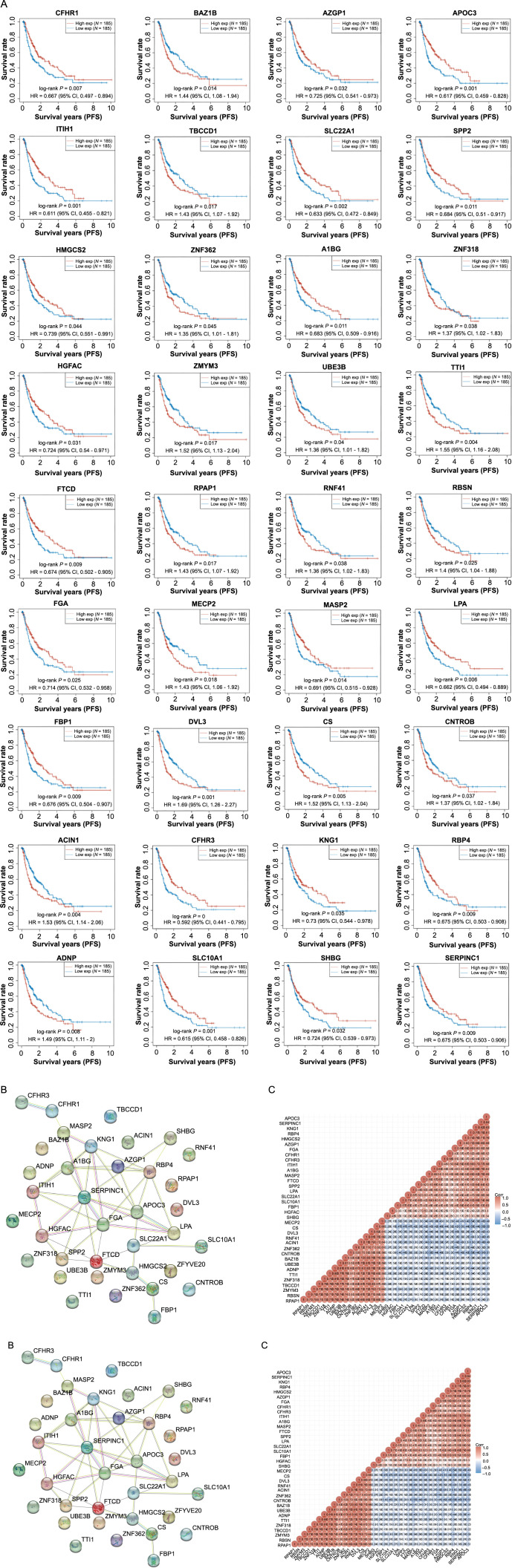
**Bioinformatics analysis of 36 genes with significant prognostic value in HCC.** (A) KM survival curves for 36 genes with significant *P* values, the horizontal axis represents the survival time, and the vertical axis represents the survival probability; (B) PPI network of 36 genes, nodes represent genes and edges represent interconnections between genes; (C) Correlation heat map of 36 genes, the horizontal and vertical axes represent genes, red represents positive correlation, and blue represents negative correlation. HCC: Hepatocellular carcinoma; KM: Kaplan–Meier; PPI: Protein–protein interactions; HR: Hazard ratio; CI: Confidence interval; Corr: Correlation; PFS: Progression-free survival.

In our analysis, we selected the top 100 upregulated and 100 downregulated DEGs for PFS rate analysis. This led to the identification of 36 genes showing a significant association with PFS (*P* < 0.05) (Figure 5A). Interestingly, higher expression of genes, such as *CHFR1*, *AZGP1*, *APOC3*, and *ITIH1*, was associated with a favorable prognosis, while the overexpression of genes like *TBCCD1*, *ZNF362*, *ZNF318*, *ZMYM3*, *UBE3B*, and *TTI1* correlated with a poorer prognosis. Further investigation into these 36 genes using the STRING database generated a PPI network consisting of 36 nodes and 45 edges (Figure 5B). The correlation analysis revealed significant positive or negative interactions among these 36 genes (Figure 5C). These findings underscore the critical role of these genes in HCC progression and potentially highlight novel prognostic markers for HCC.

### Identification of six candidate genes with prognostic value associated with HCC

**Figure 6.**
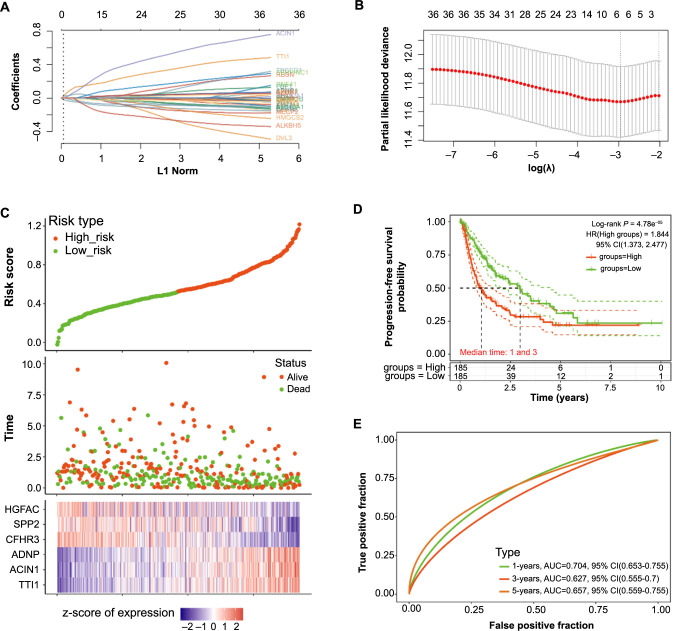
**The identification of key gene with prognostic value related to *ALKBH5* and HCC.** (A) LASSO coefficient profile of 36 genes, different colored lines represent different genes; (B) LASSO regression with ten-fold cross-validation obtained six prognostic genes using the minimum *λ* value; (C) The upper panel shows the risk score distribution of HCC patients, the middle panel represents the survival status of patients, and the lower panel is a heatmap of the expression profiles of the six prognostic genes; (D) KM survival curves showing the difference in PFS between high-risk and low-risk samples, with a median time of one and three years for the two groups of samples; (E) ROC analysis of the risk model, curves showing the true positive rate (sensitivity) versus the false positive rate (1-specificity) for different cut-off points of the risk score. LASSO: Least Absolute Shrinkage and Selection Operator; HCC: Hepatocellular carcinoma; KM: Kaplan–Meier; PFS: Progression-free survival; ROC: Receiver operating characteristic; AUC: Area under the curve; CI: Confidence interval.

We utilized the glmnet package in R to construct a LASSO Cox regression model for the 36 genes with significant *P* values. With 10-fold cross-validation, we chose 0.0521 as the minimum standard for λ (Figures 6A and 6B). Based on the nonzero coefficients of the genes, we computed the risk score for each patient as follows: (0.1124)*TTI1+(0.06)*ACIN1+(0.0402)*ADNP+(−0.0422)*CFHR3+(−0.0236)*SPP2+(−0.0094)* *HGFAC*. We used the median cutoff point obtained from the “survminer” R package to segregate the patients into high-risk (*n* ═ 185) and low-risk (*n* ═ 185) groups. As depicted in Figure 6C, patients in the high-risk group demonstrated reduced survival time compared to the low-risk group. The distribution of the six candidate prognostic genes also varied between the two groups, with the expression of *ADNP*, *ACIN1*, and *TTI1* increasing as the risk score escalated. Further, the KM survival curves indicated that the low-risk group had improved PFS compared to the high-risk group (Figure 6D). Lastly, the risk model exhibited a substantial AUC value of 0.704 in one-year survival from the ROC analysis (Figure 6E). These findings suggest that six candidate prognostic genes may be effective targets for predicting the one-year survival of HCC patients.

### Establishing *TTI1* as a key downstream gene of *ALKBH5*

**Figure 7.**
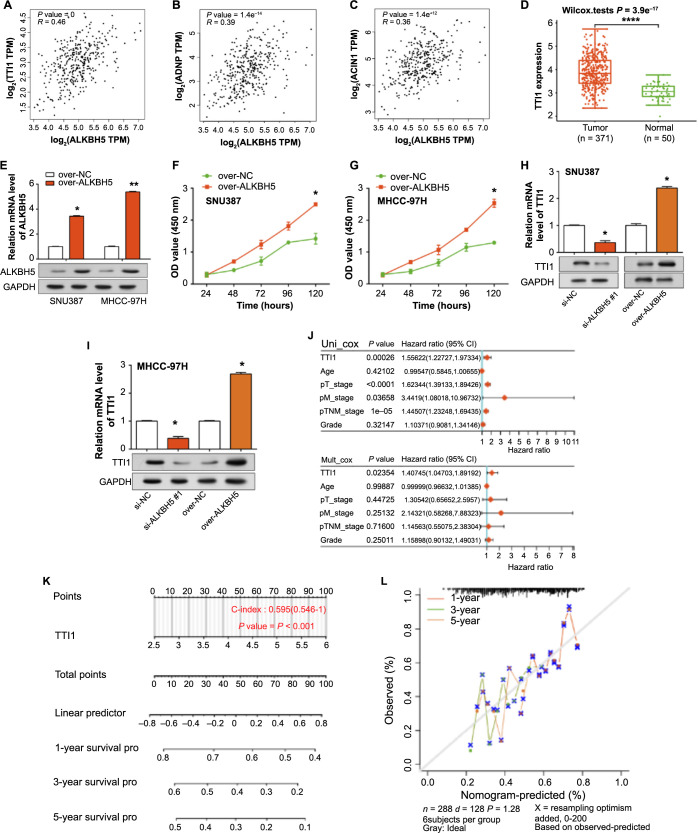
**The construction of predictive nomogram for HCC prognosis.** (A–C) Scatterplots of correlation analysis of *ALKBH5* with *TTI1*, *ADNP*, and *ACIN1* in the GEPIA database. Statistically significant *P* values and correlation coefficient *r* values are shown in the upper left corner of each graph; (D) Boxplot of *TTI1* expression levels in normal samples and HCC samples; (E) qRT-PCR and WB detection of overexpression efficiency of ALKBH5 in SNU387 as well as MHCC-97H cells; (F and G) Regulation of over-*ALKBH5* on the proliferation of SNU387 and MHCC-97H cells in CCK-8 assay; (H and I) qRT-PCR and WB detected the regulation of TTI1 expression level by knockdown or overexpression of ALKBH5 in SNU387 cells; (J) Univariate and multivariate Cox analysis of TTI1 and clinical characteristics (pT stage, pM stage, pTNM stage, grade); (K) Nomogram predicting the effect of *TTI1* on the 1-, 3-, and 5-year survival of HCC patients; (L) Calibration curve of the overall survival nomogram model in *TTI1*, the diagonal dashed line stands for the ideal nomogram and the red, yellow and grey lines stand for the observed 1-, 3- and 5-year prognosis. *P < 0.05, **P < 0.01, ****P < 0.0001. HCC: Hepatocellular carcinoma; pTNM: Pathological tumor, node, metastasis; CCK-8: Cell Counting Kit-8; qRT-PCR: Quantitative real-time polymerase chain reaction; WB: Western Blotting; TPM: Transcripts per million; OD: Optical density.

We compared the association of six candidate genes with *ALKBH5* using the GEPIA database. At a statistical significance threshold of *P* < 0.05, a significant positive connection was observed between *ALKBH5* and three genes (Figure 7A–7C). Among them, *TTI1* has the highest correlation with *ALKBH5* (*r* ═ 0.46), followed by *ADNP* (*r* ═ 0.39) and *ACIN1* (*r* ═ 0.36), therefore, we identified *TTI1* as the key downstream gene of *ALKBH5*. *TTI1* expression was shown to be considerably greater in HCC tumors than in normal tissues (Figure 7D). After *ALKBH5* was overexpressed in SNU387 and MHCC-97H cells, qRT-PCR detected a significant overexpression efficiency (Figure 7E). The results of CCK-8 showed that overexpressed *ALKBH5* significantly promoted the proliferation of SNU387 and MHCC-97H cells (Figures 7F and 7G). Furthermore, we found that the expression of *TTI1* was decreased when *ALKBH5* was knocked down, and conversely, the expression of *TTI1* was increased when *ALKBH5* was overexpressed (Figures 7H and 7I). *TTI1* was discovered as an independent predictive predictor for overall survival (OS) in HCC patients in univariate and multivariate Cox proportional hazard regression studies (Figure 7J). In clinical practice, we developed a *TTI1* nomogram to estimate 1-, 3-, and 5-year survival in HCC patients (Figure 7K). The calibration plot indicated its predictions closely aligned with actual outcomes (Figure 7L).

### *ALKBH5* combined with *TTI1* affects the proliferation, migration, and invasion of HCC cells

**Figure 8.**
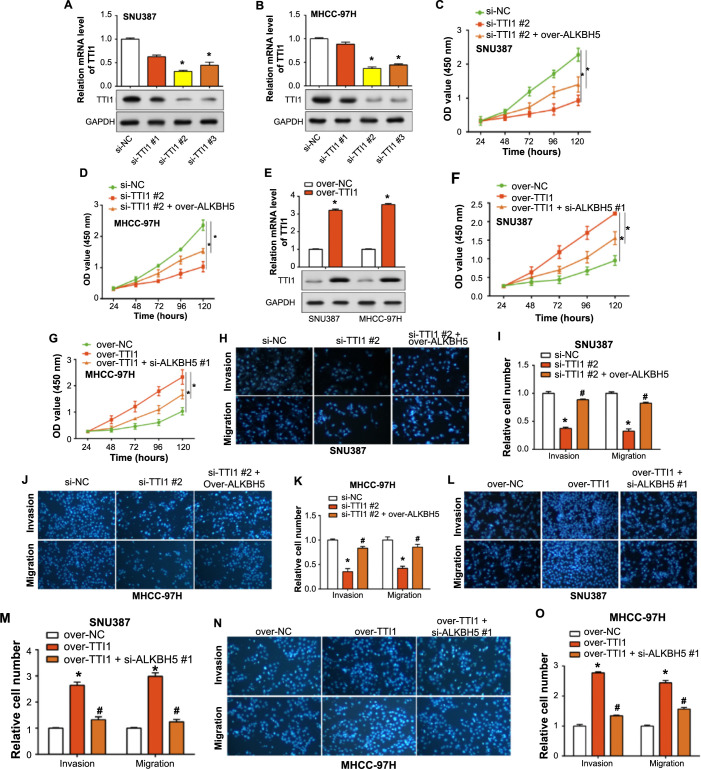
**Influence of *TTI1* and *ALKBH5* on cell proliferation, migration, and invasion in HCC cells.** (A and B) The relative expression of *TTI1* mRNA and protein in SNU387 and MHCC-97H cells transfected with si-TTI1#1, #2 and #3 was detected by qRT-PCR and WB assays; (C and D) CCK-8 test for the regulation of cell proliferation by si-*TTI1*#2 combined with over-*ALKBH5*; (E) qRT-PCR and WB assays detection of the overexpression efficiency of *TTI1* in SNU387 and MHCC-97H cells; (F and G) CCK-8 test for the regulation of over-*TTI1* combined with si-*ALKBH5*#1 on cell proliferation; (H–K) Transwell assay analysis of the regulation of si-*TTI1*#2 and over-*ALKBH5* on cell migration and invasion; (L–O) Transwell assay analysis of the regulation of over-*TTI1* combined with si-*ALKBH5*#1 on cell proliferation. *P < 0.05 vs si-NC or over-NC, #*P* < 0.05 vs si-*TTI1*#2 or over-*TTI1*. HCC: Hepatocellular carcinoma; CCK-8: Cell counting kit-8; NC: Normal control; qRT-PCR: Quantitative real-time polymerase chain reaction; OD: Optical density.

Our study investigated the impact of *TTI1* knockdown in SNU387 and MHCC-97H cells. Through qRT-PCR and WB assays, we found si-*TTI1*#2 demonstrated the most significant knockdown efficiency (Figure 8A and 8B). To elucidate the functional mechanism of *TTI1* and its upstream gene *ALKBH5* in HCC, we performed a CCK-8 assay. The results showed a decrease in cellular proliferation following the knockdown of *TTI1*. Interestingly, the overexpression of *ALKBH5* could partially mitigate the suppressive effect of si-*TTI1*#2 on cell proliferation (Figures 8C and 8D). We also induced the overexpression of *TTI1* in SNU387 and MHCC-97H cells (Figure 8E), which resulted in enhanced cellular proliferation, an effect which was diminished by the low expression of *ALKBH5* (Figure 8F and 8G). Further substantiating these observations, migration and invasion assays mirrored the trends witnessed in proliferation studies, indicating a tangible influence of *ALKBH5* and *TTI1* expression levels on the migratory and invasive potentials of HCC cells (Figure 8H–8O). Therefore, we posit that *ALKBH5*, through its regulatory action on *TTI1* expression, serves as a pivotal determinant in either promoting or inhibiting specific malignancy-associated cellular behaviors in HCC, underlining a sophisticated network of genetic interactions pivotal to cancer cell dynamics.

## Discussion

We investigated the function of *ALKBH5* in the progression and prognosis of HCC in this study. Consistent with the known risk factors for liver carcinoma, such as excessive alcohol consumption, viral hepatitis, and genetic predispositions [20, 21], our findings further confirm the complex and multifactorial nature of the etiology of HCC. Notably, we found an association between high expression of *ALKBH5* and HCC. This adds a novel dimension to the already complex landscape of HCC molecular mechanisms and provides fresh insights into potential molecular targets for therapy. Our analysis underscored the prevalence of *ALKBH5* overexpression in numerous tumor types, including HCC, thus augmenting its potential role as a pan-cancer molecular marker. Furthermore, *ALKBH5* expression levels were consistently greater in HCC clinical stage tissues compared to their normal counterparts, emphasizing its likely significance in HCC pathological development. Intriguingly, we noted minimal variation in *ALKBH5* expression across groups with differing clinical factors, suggesting that its upregulation might be a universal event in HCC, independent of individual patient characteristics. Our research serves as a seminal contribution toward comprehending the molecular underpinnings of HCC and provides robust evidence implicating *ALKBH5* as a potential therapeutic target.

The findings in vitro point to *ALKBH5* being a critical regulator of HCC cellular behavior, influencing proliferation, migration, and invasion. We observed that ALKBH5 was significantly overexpressed in HCC cell lines, especially in SNU387 cells. Knockdown of *ALKBH5* resulted in decreased cell proliferation and impaired invasion and migration capabilities, further confirming the critical role of *ALKBH5* in regulating the malignancy of HCC cells. Corroborating previous studies, *ALKBH5*, recognized as a prominent m6A demethylase [22], has been identified as a key player in a diverse array of cancers, such as breast carcinoma, stomach carcinoma, and colorectal carcinoma [23, 24]. The versatile roles of *ALKBH5* in various cancer types entail the modulation of numerous biological processes encompassing proliferation, metastasis, migration, invasion, metastasis, as well as tumor growth. Interestingly, the influence of *ALKBH5* appears to be context-dependent, with its expression level acting either as an oncogenic promoter or a tumor suppressor, depending on the type of carcinoma [25, 26]. Further supporting its multifaceted role, recent evidence also points toward an intriguing interaction between *ALKBH5* and *NEAT1* in colorectal carcinoma, proposing the ALKBH5-NEAT1 axis as a potential therapeutic target [27]. Taken together, our findings underscore *ALKBH5* as an influential factor in the pathogenesis of HCC. More thorough and in-depth research is needed, however, to elucidate the specific processes by which *ALKBH5* promotes HCC development and to prove its efficacy in clinical settings.

In the subsequent phase of our study, we performed a differential gene expression screen on HCC patients, based on *ALKBH5* expression levels. In the KEGG pathway analysis, the upregulated DEGs-enriched KEGG pathways include the Wnt signaling pathway, renal cell carcinoma, and the Hippo signaling pathway. He and Tang [28] postulated that the WNT/β-catenin signaling pathway, a highly conserved and tightly controlled molecular mechanism, governs cellular differentiation, proliferation, and embryonic development. Notably, there is increasing evidence that abnormalities in WNT/β-catenin signaling contribute to the progression and development of liver carcinoma, which contains HCC and CHOL [29, 30]. In addition, when Takebumi Usui et al. studied cases of renal cell carcinoma liver metastases, they found that many patients with renal cell carcinoma after surgical resection would develop in the direction of liver carcinoma [31]. The Hippo pathway was found to be a critical regulator of liver size, metabolism, development, regeneration, and homeostasis in genetic studies on murine livers conducted by Driskill and Pan. Abnormalities in this pathway may contribute to common liver diseases like liver carcinoma and fatty liver disease [32]. Besides, the downregulated DEGs are abundant in Tryptophan metabolism, Retinol metabolism, Pyruvate metabolism, Histidine, and Glutathione metabolism. This underlines the intricate interplay between liver carcinoma and functional molecular metabolism within the human body. For instance, research by Han et al. [33] delineates an age-related metabolic imbalance in the liver involving glycerophospholipids, arachidonic acid, histidine, and linoleic acid. In summary, our exploration of the roles of various metabolic and signaling pathways provides valuable insights into the molecular landscape of HCC, highlighting the potential for targeting these specific pathways for therapeutic intervention.

Through PFS survival, PPI, correlation, LASSO, Cox, and other prognostic value analyses, we identified six key genes (*HGFAC*, *SPP2*, *CFHR3*, *ADNP*, *ACIN1*, and *TTI1*) associated with HCC prognosis. Following that, we used the GEPIA database to determine the connection between *ALKBH5* and these genes, finally identifying *TTI1* as the most significant prognostic gene. *TTI1*, or TELO2 Interacting Protein 1, plays a vital role in various biological processes, yet remains relatively understudied. Existing literature suggests that *TTI1* is involved in multiple metabolic pathways and in the activation of mTORC1 signaling, which promotes cell growth [34, 35]. For instance, *TTI1* has been shown to facilitate survival in multiple myeloma via the mTORC1 pathway [36]. Rao et al. [37] revealed the role of *TTI1* in binding ATM and DNA-PKcs, triggering the activation of p-53 and S-15 phosphorylation pathways to initiate cancer cell death programs. Furthermore, research on colorectal cancer by Xu et al. [38] indicated higher *TTI1* expression in tumor tissue relative to adjacent normal tissue, demonstrating its critical role in colorectal cancer proliferation. Nevertheless, the influence of *TTI1* on the development of liver carcinoma is still not clear.

We conducted a thorough study to investigate the function of *TTI1* in HCC, and the findings underscored the importance of both *TTI1* and *ALKBH5* in the development of HCC. TTI1 was discovered to be considerably overexpressed in HCC tumors as compared to normal tissues. Notably, *ALKBH5* overexpression was seen to significantly enhance SNU387 cell proliferation, an effect inversely mirrored by *TTI1* under-expression. *TTI1* also emerged as an independent prognostic indicator for OS in HCC patients, prompting us to construct a predictive *TTI1* nomogram with high consistency between predicted and actual survival rates. The interaction between *TTI1* and *ALKBH5* revealed their influence on HCC cell growth. Downregulation of *TTI1* suppressed cell proliferation, migration, and invasion, a result that was partially counteracted by *ALKBH5* overexpression. *TTI1* overexpression, on the other hand, enhanced cell proliferation, migration, and invasion but was inhibited by reduced *ALKBH5* expression. Altogether, these findings underscore a potential regulatory role of *ALKBH5* in HCC progression via modulation of *TTI1* expression, illuminating novel avenues for potential therapeutic strategies.

## Conclusion

Our findings confirm the characterization of *ALKBH5* and *TTI1* as oncogenes in HCC, emphasizing their potential as novel markers in HCC. Through bioinformatics analysis and cellular experiments, we elucidated that the interaction between ALKBH5 and TTI1 significantly affects the proliferation, migration, and invasion of HCC cells, suggesting that ALKBH5 may exert a key regulatory influence on HCC progression by regulating TTI1 expression. These findings greatly advance the current understanding of HCC and pave the way for innovative directions for future research.

## Data Availability

The datasets used and/or analyzed during the current study are available from the corresponding author on reasonable request.
